# Early Development of Atherosclerotic Plaques in the Coronary Arteries after Radiotherapy for Breast Cancer (BACCARAT Study)

**DOI:** 10.3390/jcdd10070299

**Published:** 2023-07-12

**Authors:** Manoj Kumar Honaryar, Rodrigue Allodji, Gaelle Jimenez, Mathieu Lapeyre, Loic Panh, Jeremy Camilleri, David Broggio, Jean Ferrières, Florent De Vathaire, Sophie Jacob

**Affiliations:** 1 INSERM U 1018, CESP, Radiation Epidemiology Team, 94800 Villejuif, France; mkhonaryar@gmail.com (M.K.H.); rodrigue.allodji@gustaveroussy.fr (R.A.); florent.devathaire@gustaveroussy.fr (F.D.V.); 2 Gustave Roussy, Research Department, 94800 Villejuif, France; 3 University Paris-Saclay, 94800 Villejuif, France; 4 Department of Radiation Oncology (Oncorad), Clinique Pasteur, 31076 Toulouse, France; gjimenez@clinique-pasteur.com; 5 Department of Radiology, Clinique Pasteur, 31076 Toulouse, France; mlapeyre@rx-infomed.com (M.L.); jcamilleri@clinique-pasteur.com (J.C.); 6 Department of Cardiology, Clinique Pasteur, 31076 Toulouse, France; panh.loic@gmail.com; 7 Department of Dosimetry, PSE-SANTE/SDOS/LEDI, Institute for Radiation Protection and Nuclear Safety (IRSN), 92260 Fontenay-aux-Roses, France; david.broggio@irsn.fr; 8 Department of Cardiology and INSERM UMR 1295, Rangueil University Hospital, 31400 Toulouse, France; jean.ferrieres@univ-tlse3.fr; 9 Laboratory of Epidemiology, PSE-SANTE/SESANE/LEPID, Institute for Radiation Protection and Nuclear Safety (IRSN), 92260 Fontenay-aux-Roses, France

**Keywords:** breast cancer, radiotherapy, coronary atherosclerotic plaques, contrast-enhanced CT angiography

## Abstract

Background—Radiotherapy (RT) for breast cancer (BC) can lead to an increased risk of coronary artery disease several years after RT. The aim of this study was to evaluate the development of overall, non-calcified and calcified atherosclerotic plaques over 2 years after BC for RT and associations with cardiac exposure. Methods—The study included 101 left- or right-sided BC patients treated with RT without chemotherapy. A coronary CT angiography was performed before and 2 years after RT. Plaque development thorough the entire coronary network was defined as an increased number of plaques. Cardiac exposure was quantified with mean doses to the heart, left ventricle, and coronary arteries. Logistic regression models were used to assess association with doses. Results—At inclusion, 37% of patients had plaques, increasing to 42% two years after RT. Overall plaque development was observed in seven patients: five with calcified plaque development and four with non-calcified plaque development. The risk of overall plaque development was significantly associated with doses to the Left Main and Circumflex coronary arteries (OR at 1 Gy = 2.32, *p* = 0.03 and OR at 1 Gy = 2.27, *p* = 0.03, respectively). Specific analyses for calcified and non-calcified plaque development showed similar results. Conclusion—Our study suggests an association between coronary arteries exposure and the risk of developing both calcified and non-calcified atherosclerotic plaques over 2 years after BC RT. Trial registration number: NCT02605512.

## 1. Introduction

Breast cancer is the most common type of cancer and the leading cause of cancer death worldwide among women [1]. In the therapeutic arsenal to treat breast cancer, radiation therapy (RT) is a cornerstone, with more than 75% of patients undergoing therapeutic radiation [2,3,4]. Although, it generally provides a clear improvement in local tumor control and significantly reduces the risk of cancer-related mortality several years after treatment, it also results in irradiation of the heart due to its anatomical position. With the improvement of cancer management and the improvement in the survival of women treated for BC, enhancing knowledge on RT-related adverse cardiovascular effects that may occur several years or decades after RT has become a real challenge to maintain the patients quality of life [5].

It has been shown that RT for breast cancer can lead to an increased risk of coronary artery disease which starts a few years after the RT and continues for at least for two decades [6,7,8,9]. Atherosclerosis, the underlying principal cause of these diseases, results from the progression of atheromatous plaques in the coronary arteries and remains asymptomatic for many years due to sufficient functional reserve of these arteries until significant occlusion occurs [10]. Depending on their composition, atheromatous plaques can be either non-calcified or calcified [11]. Coronary artery calcification (CAC) scoring based on non-contrast enhanced cardiac CT is recognized as a good predictor of coronary artery disease, even for early detection of coronary artery disease in asymptomatic individuals [12]. For patients treated with RT, previous studies observed associations between cardiac exposure and CAC score [13,14] or CAC increase [15,16]. However, asymptomatic individuals with zero CAC score may have not yet developed detectable, calcified coronary plaque but may have early stages of plaque and non-calcified plaques. A longtime before the clinical manifestation of coronary atherosclerotic disease occurs, the atherosclerotic plaques (both calcified and non-calcified) can be well-identified and measured by contrast-enhanced coronary CT angiography [17,18]. The interest in evaluating both calcified and non-calcified plaques has been illustrated in a large study which showed that patients with non-calcified plaques had a three times higher risk of coronary events than patients with calcified ones [19]. Diffuse coronary plaque can be present without detectable CAC score, illustrating the interest for sensitivity to evaluate coronary atherosclerotic plaques in addition to the CAC score [20]. To our knowledge, no study was conducted to investigate atherosclerotic plaques via coronary CT angiography in breast cancer patients treated with RT.

Long before the potential occurrence of radiation-induced coronary artery disease, a precise evaluation of atherosclerotic plaques, both calcified and non-calcified, quantifiable using coronary CT angiography, could allow for a better understanding of the early stages of radiation-induced atherosclerosis and the possible implementation of preventive measures. The aim of this study was to assess the impact of breast cancer RT on development of atheromatous plaques (both calcified and non-calcified) occurring within 2 years of RT and estimate the association between cardiac exposure and the risk of developing these plaques.

## 2. Materials and Methods

### 2.1. Study Design and Population

This monocentric prospective cohort study included 118 female BC patients of the Clinique Pasteur, Toulouse, France, from October 2015 to December 2017, aged 40 to 75 years old, mainly with left unilateral BC and in a smaller proportion with right-sided unilateral BC, without history of severe cardiovascular disease such as coronary artery disease including acute myocardial ischemia or infarction [21]. Patients with renal failure, allergies to iodinated contrast injection, and pregnancy were also excluded. All patients were treated with adjuvant three-dimensional conformal radiation therapy (3D-CRT) after breast conserving surgery or mastectomy, without chemotherapy. Patients were followed from baseline before RT to 2 years after RT. Patient’s medical history was collected at baseline and physical examinations, in particular coronary CT angiography (CTCA), were performed by the cardiologists and radiologists, respectively, during the programmed consultations at baseline and 2 years after RT. Five patients withdrew consent and twelve patients had missing data (either for CTCA measurements or cardiac exposure measurements). Finally, the study population presented here consisted of 101 patients with complete data on coronary artery atherosclerotic plaques at baseline and 2 years after RT, as well as cardiac radiation dosimetry data.

This study was approved by the French Southwest Ethics Committee for Protection of Persons (ID: CPP2015/66/2015-A00990-69) and by the National Agency for Medical and Health products Safety (Reference: 150873B-12). Written informed consent form was obtained from all the patients participating in our study.

### 2.2. Radiotherapy

All the BC patients were treated with 3D-CRT with or without irradiation of supraclavicular or internal mammary lymph nodes after initial surgical treatment by either radical mastectomy or breast conserving—lumpectomy. After raising both arms above thier heads, patients were positioned on a breast board. Planning target dose was 50 Gy delivered in 5 weeks with 25 daily doses of 2 Gy, or the second schema of 47 Gy delivered in 5 weeks with 20 daily doses of 2.35 Gy for the patients treated between January 2016 and May 2016. This second schema of hypo-fractioned dose administration decision was made for logistic purposes because of limited access to one of the 3D-CRT machines it was necessary to slightly limit the number of sessions per patient. Six MV photons were used for most of the study participants. However, for a few cases with big breast sizes, 25 MV additional photons were delivered. In addition, on a case-by-case basis, a boost of 12 to 12.5 Gy (with 4 to 5 fractions of 3 or 2.5 Gy) could be applied to the tumor site with electron/photons beams, with energies ranging from 6 MeV to 18 MeV or 6 MV. To conduct whole heart dose calculations, the Eclipse™ Treatment planning system (TPS) and the integrated software Analytical Anisotropic Algorithm (AAA v13.6) (Varian Medical System, Palo Alto, CA, USA) were used. The resulting doses of all irradiated breast volumes were taken into account. For each patient, the RT was planned in a manner where the distribution was normalized and optimized based on the International Commission on Radiation Units and Measurements (ICRU) point of reference for the breast and to obtain QUANTEC dose constraints to the organs at risk, e.g., the heart [22]. Deep inspiration breath hold (DIBH) was only used for patients treated for left-sided BC who had a heart very close to the anterior chest wall or for dose constraints achieved according to Clinic Pasteur radiotherapy protocols (mean heart dose < 5Gy and V25Gy < 10%).

### 2.3. Radiation Doses

The methods used for the evaluation of radiation doses distribution to the whole heart, left ventricle, left main coronary artery (LMCA), left anterior descending artery (LAD), circumflex artery (CX), and right coronary artery (RCA) in BACCARAT patients are described elsewhere [21,23]. In short, the Dose–Volume Histogram (DVH) for the heart was produced by the RT department of the Clinic Pasteur. The delineation of the cardiac sub-structures was performed manually taking advantage of patients’ Coronary Computed Tomography Angiography. DVHs for additional cardiac sub-structures were generated with ISOGray TPS by the dosimetry department of IRSN in collaboration with the Clinic Pasteur RT department by using the 3D dose matrix created during treatment planning. From the DVHs, the several absorbed dose metrics for cardiac structures and coronary arteries were calculated. For the present analysis, Dmean (in Gy), the volume-weighted mean dose, was considered for each delineated cardiac structure, i.e., whole heart, left ventricle, and coronary arteries including LMCA, LAD, CX, and RCA.

### 2.4. Coronary CT Angiography

#### 2.4.1. Acquisition

Baseline and follow-up cardiac CT scans were performed with SIEMENS dual-source CT (SOMATOM FLASH definition, Siemens Imaging system ^®^, Erlangen, Germany) with contrast injection either XENETIX 350 or OPTIJECT 350. Axial CT images were reconstructed with a slice thickness of 3 mm with retrospective ECG gating. In order to enhance scan quality of coronary CT angiography and to limit coronary CT artefacts, several measures were applied, including heart rate control with injection of β-blockers (if no contraindication), breath-hold instructions, optimal cardiac cycle phase reconstruction.

#### 2.4.2. Analysis

**Figure 1:**
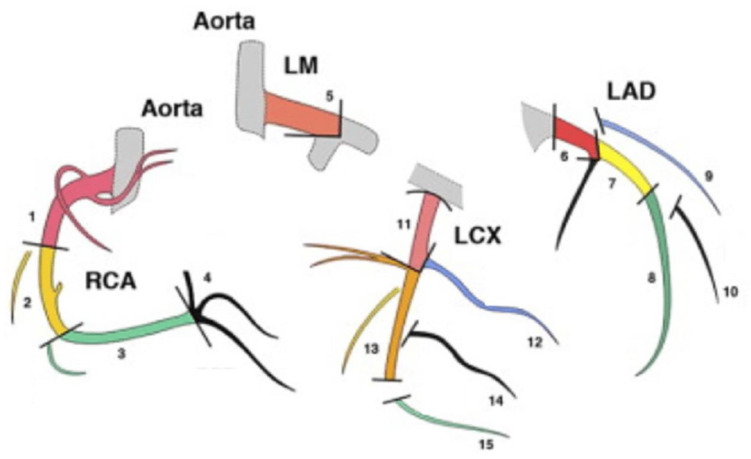
Description of the segments of the coronary arteries network. LM: Left main coronary artery; LAD: left anterior descending artery; LCX: left circumflex artery; RCA: right coronary artery (adapted from [21]).

Coronary reconstructions blinded to patient name and study date were transferred to an offline workstation and curved multi-planar reformations generated using CT comprehensive cardiac intellispace portal Philips NV software. The extent of atherosclerotic plaque burden at both baseline and follow-up was assessed for the entire left main (LM), the left anterior descending (LAD), left circumflex (LCX), and right (RCA) coronary artery according to the arterial tree segmentation presented in Figure 1. 

An experimented radiologist (ML, 15 years of experience in cardiovascular imaging), blinded to time of scanning (baseline/ follow-up) and laterality of RT, determined the presence of calcified or non-calcified plaque in all evaluable segments. Calcified plaque was defined by any structure distinct from the vessel lumen within the artery wall with a CT attenuation greater than 130 HU. The presence of any calcification within the corresponding segment rendered the segment as calcified. Non-calcified plaque was defined by a structure assigned to the coronary artery wall with CT attenuation above the surrounding tissue, but below that of the contrast enhanced lumen without any calcified plaque being present. For this analysis, we only considered two categories of plaques: either calcified or non-calcified. Despite some plaques being partially calcified and partially non-calcified, we categorized them as calcified plaques.

#### 2.4.3. Definition of Endpoints

Plaque development thorough the entire coronary network and for each coronary artery (LM, LAD, CX and RCA) was defined at patient-level as follows:

- Any plaque development: the number of segments containing any plaque had increased by at least 1 between baseline and RT + 2 years.
- Calcified plaque development: the number of segments containing calcified plaque had increased at least by 1 between baseline and RT + 2 years.
- Non-calcified plaque development: the number of segments containing non-calcified plaque had increased at least by 1 between baseline and RT + 2 years.

### 2.5. Clinical Covariates

At baseline, we collected information on cardiovascular risk factors known to have a potential impact on coronary atherosclerosis development and progression for all study participants. These covariates included age at start of radiotherapy, body mass index, smoking (defined by current or previous daily cigarette use), hypertension (defined by a systolic blood pressure of ≥140 mmHg or diastolic of ≥90 mmHg or current anti-hypertensive treatment), diabetes (defined by treatment with hypoglycemic medication), dyslipidemia (defined with lipid medication), cardiovascular treatment and statins treatment (both defined as ongoing treatments at baseline). In addition, we considered hormonal therapy use which are common in BC patients. 

### 2.6. Statistical Analyses

The descriptive analyses were expressed as means and standard deviations (SD) for quantitative variables, absolute numbers (n), and relative percentages (%) frequencies for qualitative variables. The comparisons were performed using the non-parametric Wilcoxon test for continuous data and Chi2 for categorical data (proportions and percentages). The associations between the endpoints of plaque progression and radiation and non-radiation factors were analyzed in a univariate analysis based on logistic regressions (odds ratios (ORs), 95% confidence intervals (CI), *p*-values). Cardiac radiation exposure factors included mean doses (Dmean) of the heart, the left ventricle, and coronary arteries. Non-radiation cardiac risk factors included covariates known to have a potential impact on coronary atherosclerosis development and progression such as age, body mass index (BMI), smoking, hypertension, diabetes, dyslipidemia, endocrine therapy, cardiovascular treatment, and statins use. Multivariate analysis combining radiation dose with other preliminary identified cardiac risk factors (with *p*-value in univariate analysis <0.20) was initially planned in order to evaluate the combined effect of radiotherapy and other risk factors. However, due to small sample size, it was not performed. The analyses of the data were conducted using software; Stata 14.2 STATA corp, and SAS version 9.4. *p*-values < 0.05 (two-sided) were considered statistically significant.

## 3. Results

### 3.1. Baseline Characteristics of the Study Population

Baseline demographics and cardiac dosimetry for the 101 patients (85% left-sided BC and 15% right-sided BC) are presented in Table 1. The mean age of patients was 58 ± 8 years, 7% of patients had diabetes, 15% had hypertension, 45% of the patients were current or former smokers, 7% were treated with statins. Most patients received hormonal therapy (76%), either anti-aromatase or tamoxifen). The mean heart and mean left ventricle doses were, respectively, 2.5 ± 1.5 Gy and 5.2 ± 3.7 Gy. For coronary arteries, the most exposed one was the LAD with 13.1 ± 9.0 Gy, followed at a lower level by the CX with 1.4 ± 0.9 Gy. 

### 3.2. Atherosclerotic Plaque Description at Baseline and 2 Years after RT

At baseline, a total of 1404 out of 1414 (101 × 14) segments could be analyzed thorough the entire coronary network: 101 segments for LM, 604 segments for LAD, 302 segments for CX, and 397 segments for RCA. Among them, 128/1404 segments (9.1%) contained atherosclerotic plaque: 4/101 (3.9%) were located in LM, 79/604 (13.1%) were located in LAD, 15/302 (4.9%) in CX, and 28/397 (7.0%) in RCA. At patient-level, it corresponded to 37 patients (36.6%) with atherosclerotic plaque (27.7% with non-calcified plaques, 22.8% with calcified plaques): 4.0% of patients had plaques in LM, 32.7% in LAD, 9.9% in CX, and 15.8% in RCA (Table 2), and the mean number of plaques per patient was 1.24 ranging from 0 to 12.

Two years after RT, a total of 1407 segments could be analyzed, including 134 segments (9.5%) with any atherosclerotic plaque: 4/100 (4.0%) were located in LM, 85/606 (14.0%) were located in LAD, 16/302 (5.2%) in CX, and 29/399 (7.2%) in RCA. At patient-level, it corresponded to 42 patients (41.6%) with atherosclerotic plaque: 4.0% of patients had plaques in LM, 38.6% in LAD, 9.9% in CX, and 16.8% in RCA (Table 2) and the mean number of plaques per patient was 1.32 ranging from 0 to 12. 

None of the observed increases in number of plaques from baseline to RT + 2 years either at segment-level or patient-level were statistically significant. Evaluation of these increases yielded to the identification of patients with the endpoints of plaques development. Patients with a higher number of plaques at RT + 2 years than at baseline were defined with plaque development: seven patients presented atherosclerotic plaque development (zero for LM, six for LAD, zero for CX, and one for RCA), five patients presented calcified plaque development, and four patients with non-calcified plaque development (Table 2). Among the seven patients with plaque development, five patients had no plaques at baseline, one patient had one plaque at baseline and two plaques at RT + 2 years, and one patient had two plaques at baseline and three plaques at RT + 2 years. Among the five patients with calcified plaque development, three patients with no plaque at baseline (either calcified or non-calcified) developed calcified plaque after RT, one patient with calcified plaques at baseline had an increased number of calcified plaques after RT, one patient with only non-calcified plaques at baseline became calcified after RT. Moreover, all patients with plaque development were left-sided BC patients. A detailed description of plaque development at segment-level is presented in Appendix A Table A1 for the seven patients with plaque development. It allowed us to observe that two patients with already existing plaques at baseline, developed additional plaques 2 years after RT, and five patients with no plaques at baseline developed plaques, mainly located in the LAD, after RT. 

### 3.3. Association between Radiation Doses and Atherosclerotic Plaque Development

**Figure 2:**
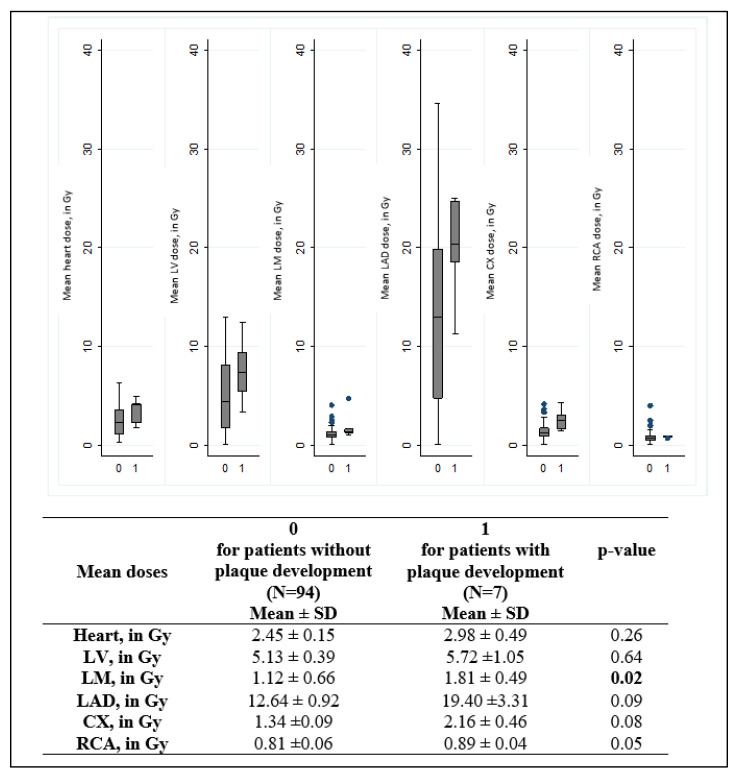
Comparison of cardiac doses according to the overall coronary plaque progression status (0 for no plaque progression; 1 for plaque progression). LM left main common coronary artery, LAD: left anterior descending artery, CX: circumflex artery; RCA: right coronary artery. The central value of the box indicates the means, the borders of the box indicate the quartiles (25th and 75th), and the extremities indicate the minimum and maximum values. *p*-value: results of Wilcoxon test to compare dose distributions.

A comparison of dose distributions to the heart, left ventricle, and coronary arteries between the groups of patients with or without plaque development (Figure 2) allowed us to observe slightly higher doses for patients with plaque development, but none of these differences reached statistical significance, except for dose to the LM: 5.7 Gy vs. 5.1 Gy (*p* = 0.02).

Among non-radiation covariates, diabetes was the only one with a significant difference between both groups (5.3% in patients without plaque development vs. 28.6% in patients with plaque development, *p* = 0.02) but it was based on a very limited number of patients (five vs. two) (Table 3). 

The association between the risk plaque development and cardiac radiation exposure was further investigated with univariate logistic regressions. Multivariable analysis combining radiation dose with diabetes (with *p*-value in univariate analysis <0.20) was not performed, due to small sample size. The risk of any atherosclerotic plaque development was significantly associated with doses to the LM (OR at 1 Gy = 2.32 [1.08–4.97]; *p* = 0.03) and dose to the CX (OR at 1 Gy = 2.27 [1.09–4.74]; *p* = 0.03). For other doses, including heart, left ventricle, LAD, and RCA, associations were positive (OR > 1), but none were significant (Table 4). 

Specific analysis for calcified and non-calcified plaque development showed similar and consistent results with significant associations in the same order as any plaque development for doses to LM and CX. Due to the very limited number of events, we did not perform multivariable analyses. Four analysis of correlations of different cardiac substructures doses (Appendix A Table A2) allowed us to observe that the heart, LV, and LAD doses were well correlated with each other, whereas LM and CX doses were well correlated with each other. The RCA dose remained poorly correlated with other cardiac structures.

## 4. Discussion

In this prospective study of 101 BC patients treated with 3D-CRT without chemotherapy, seven patients (all left-sided BC), followed for 2 years, developed coronary atherosclerotic calcified and/or non-calcified plaques, characterized by an increase in the number of plaques from baseline to RT + 2 years, mainly located in the LAD. We observed a significant dose–response relationship between atherosclerotic plaque development and mean doses to the left main and circumflex coronary arteries. This association was also observed by analyzing specifically calcified or non-calcified plaque.

Traditional stress tests used to diagnose coronary artery disease only detect flow-limiting stenosis and may miss early coronary atherosclerosis. In our study, which aimed to investigate early coronary atherosclerosis arising within 2 years after RT for BC, all patients were asymptomatic, and we only observed one patient with severe stenosis (>50%) after RT, illustrating the interest of using non-invasive imaging of coronary plaques to enhance the understanding of early atherosclerosis and its pathogenesis. Coronary CT angiography gives detailed information about non-obstructive atherosclerosis, useful to evaluate the risk of acute coronary syndromes which are frequently caused by non-obstructive coronary plaques [24]. Coronary atherosclerotic plaques can be classified as calcified, non-calcified, or mixed [11]. Previous studies, including ours, showed an association between cardiac exposure and increased coronary artery calcium score [6,17,25]. Although the coronary artery calcium score serves as a surrogate for total coronary atherosclerotic burden, and correlates with risk of mortality, [26] it does not consider non-calcified plaque, which may be more relevant for risk assessment [27,28]. Non-calcified coronary plaques can even be found in individuals who have zero coronary calcium scores and literature shows that non-calcified atherosclerotic plaques are more metabolically active than heavily calcified plaques and are associated with increased risk of acute coronary syndromes [29,30]. 

The study presented here, is the first study on BC patients treated with RT that investigated coronary atherosclerotic plaques classified as calcified or non-calcified [11]. We observed not only the apparition of calcified plaques in five patients (which can be due to the aging process as well as stabilization of non-calcified plaques during this two years of the follow-up period), but also the apparition of non-calcified plaques in four patients. Plaque instability is related to the degree of ongoing inflammation and plaque composition [31]. Non-calcified atherosclerotic plaques are associated with increased risk of acute coronary syndromes due to the early rupture and shedding of the plaques in contrast to calcified plaques which are considered stable [29]. Non-calcified coronary plaque and the total plaque burden of the coronary arteries, even of only one segment, are associated with an increased risk of cardiovascular events [32].

The pathophysiology of radiation-induced coronary artery disease is considered to be a result of accelerated atherosclerotic disease of the coronaries [33]. Among the five patients with an increased number of calcified plaques, three patients had no plaque at baseline. It raises the question whether non-calcified plaques may have appeared after RT but before RT + 2 years and rapidly calcified in time. Calcification enters later during the inflammatory process to stabilize the plaque and avoid shedding [34]. It might take five years for calcification to occur in coronary arteries [35]. Our results may thus be coherent with the concept that the RT accelerates the process of already existing atheromatous plaques [36]. In addition, the four patients with non-calcified plaques development had no plaque at baseline. Our results, thus, also suggest that RT not only has impact on previously existing plaques but may also initiate the apparition of (non-calcified) plaques.

In our study, 37 (36.6%) patients had atherosclerotic plaques at baseline, and 42 (41.6%) patients at the end of the follow-up 2 years after RT. In the general population, data on the prevalence of the presence of atherosclerotic plaques were published from a large Swedish study of 30,000 individuals [37]. The prevalence of atherosclerotic plaque was 29.3% for women aged 55–59 years old and 40.1% for women aged 60–64 years old. In our study, the mean age at baseline was 58.4 years, and we observed 36.6% with any atherosclerotic plaque at baseline, reaching 41.6% two years after RT. These prevalences are quite consistent with the Swedish general population study. We observed development of atherosclerotic plaques in a small subset of breast cancer after radiotherapy (7 patients). Regarding the development of plaques with a follow-up of 2 years, a study was performed in patients who presented with acute chest pain to the emergency room but had initially no evidence for acute coronary syndrome [38]. A 12.7% increase in the mean number of segments containing any plaque was observed in this specific population. Age > 64 years was the most important risk factor of plaque progression, and at a lower-level hypertension, diabetes, statin use, history of CAD. In our study, 6.9% of patients had an increased number of plaques at the 2 year follow-up, and we found no significant difference in either age or several other risk factors between patients with plaque development and patients without plaque development, except for diabetes. In our study, based on few cases, the development of atherosclerotic plaques was thus not associated with age and other factors. However, we observed a dose–response relationship with coronary artery exposure, which is an important argument in the plausibility for causal relationship between RT and plaque development.

For both type of plaques, mainly localized in the LAD, an association between mean doses to the left main and circumflex coronary was observed. However, no significant association with mean dose to the LAD was observed. By analyzing correlations between mean doses to different cardiac substructures, we found that LM and CX were highly correlated (r^2^ = 0.73) together, but poorly correlated with other cardiac structure such as the whole heart, left ventricle, and LAD (r^2^ < 0.52). This can explain why both LM and CX were both linked to plaque development. The absence of association with mean LAD dose is surprising but could suggest that mean LAD doses may present limits for detailed investigation of plaques along the LAD and lack of precision to consider heterogeneity of dose from the upper part to the lower part of the LAD. LM or CX may be better proxies of average exposure than LAD dose even if plaques are located in LAD. For future studies, it could be interesting to have a very detailed dosimetry at the scale of segments of these coronary arteries to evaluate the segmental heterogeneity. 

### Limitations 

Our results are limited by the small size of the population and the small number of patients with the event of interest. However, this is the first study which investigated early coronary atherosclerotic plaques development, both calcified and non-calcified, based on coronary CT angiography performed at baseline before RT and 2 years after RT in a population of BC patient treated with RT without chemotherapy. We did not evaluate the impact of RT and radiation dose on the degree of stenosis. We observed that only one left-sided BC patient developed clinically significant coronary artery stenosis (>50%) two years after RT. With a longer follow-up, we could hypothesize that additional patients could develop clinically significant stenosis, requiring specific medical care. None of the classic cardiovascular risk factors was significantly associated with our outcome, except diabetes, but due to a very limited number of events, we could not conduct multivariable analyses. As a consequence, we could not evaluate the combined effect of radiotherapy and other risk factors. Information on statin use was collected at baseline but not after RT; therefore, we could not analyze the possible impact of statins or other cholesterol lowering drugs initiated after the RT that could have influenced plaque development. Thus, further studies are needed, with larger sample sizes, such as the MEDIRAD EARLY-HEART study [39] and longer follow-up to further explore in detail the initiation and progression of the coronary atherosclerotic plaques.

## 5. Conclusions

During the course of 2 years, coronary atherosclerotic plaque development can be observed in BC patients treated with RT and is associated with radiation exposure to coronary arteries. Both calcified and non-calcified plaques development can be observed, suggesting that RT-induced cardiac radiation exposure accelerates the process of already existing atheromatous plaques (calcified plaques) but may also initiate the apparition of de novo plaques (non-calcified). Further studies remain needed to confirm these results.

## Figures and Tables

**Table 1:** Baseline characteristics of the population of 101 BC patients.

Variables	Mean ± SD or N (%)
Age (years)	58.4 ± 0.8
**Cancer treatment**	
Laterality	
*Left-sided BC*	84 (83.2)
*Right-sided BC*	17 (16.8)
Histology	
in situ	19 (18.8)
*Invasive*	82 (81.2)
Type of surgery	
*Conservative*	94 (93.1)
*Mastectomy*	7 (6.9)
Protocol of radiotherapy	
*50 Gy (25 x 2Gy)*	76 (75.3)
*47 Gy (20 x 2.35Gy)*	25 (24.7)
Regional lymph nodes irradiation (Supraclavicular and/or Internal mammary Chain)	28 (27.7)
Boost	92 (91.1)
Adjuvant endocrine therapy	77 (76.2)
*Anti-aromatase*	45 (58.4)
*Tamoxifen*	32 (41.6)
**Cardiovascular risk factors**	
BMI (kg/m^2^)	24.4 ± 0.4
Hypercholesterolemia	33 (32.7)
Diabetes	7 (6.9)
Cardiovascular treatment	15 (14.9)
*Statines (medication)*	6 (5.9)
**Cardiac dosimetry**	
Whole heart (Gy)	2.49 ± 1.45
Left ventricle (Gy)	5.17 ± 3.72
LM (Gy)	1.17 ± 0.71
LAD (Gy)	13.11 ± 9.02
CX (Gy)	1.40 ± 0.89
RCA (Gy)	0.82 ± 0.53

BMI: Body mass Index; LM: Left main coronary artery; LAD: left anterior descending artery; CX: left circumflex artery; RCA: right coronary artery.

**Table 2:** Description of atherosclerosis plaques at patient-level at baseline and 2 years after RT.

Location of Plaques	Number of Patients with Plaque at Baseline N (%)	Number of Patients with Plaque at RT + 2 Years N (%)	*p*-Value *	Number of Patients with Plaque Development N (%)
**Any plaque**				
Entire coronary arterial network	37 (36.6)	42 (41.6)	0.90	7 (6.9%)
LM	4 (4.0)	4 (4.0)	1.00	0 (0%)
LAD	33 (32.7)	39 (38.6)	0.38	6 (5.9%)
CX	10 (9.9)	10 (9.9)	1.00	0 (0%)
RCA	16 (15.8)	17 (16.8)	0.85	1 (1.0%)
**Non-calcified plaque**				
Entire coronary arterial network	28 (27.7)	31 (30.7)	0.64	4 (3.9%)
**Calcified plaque**				
Entire coronary arterial network	23 (22.8)	27 (26.7)	0.51	5 (4.9%)

BMI: Body mass Index; LM: Left main coronary artery; LAD: left anterior descending artery; CX: left circumflex artery; RCA: right coronary artery. * based on Mac Nemar test for comparison of the proportion of patients with plaque at baseline vs. RT + 2 years.

**Table 3:** Comparison of non-radiation covariates between patients without and with plaque development.

Covariates	Patients without Plaque Development (N = 94) Mean ± SD or N (%)	Patients with Plaque Development (N = 7) Mean ± SD or N (%)	*p*-Value
Age (years)	58.21 ± 0.86	61.43 ± 1.39	0.32
Body mass index (kg/m^2^)	24.44 ± 0.43	24.54 ± 1.27	0.75
Hypertension	14 (14.9)	1 (14.3)	0.96
Smoking status (Former or current)	43 (45.8)	3 (42.9)	0.80
Hypercholesterolemia	31 (32.9)	2 (28.6)	0.81
Diabetes	5 (5.32)	2 (28.6)	0.02
Cardiovascular treatment	14 (14.9)	1 (14.3)	0.97
Statines (medication)	5 (5.3)	1 (14.3)	0.33
Adjuvant endocrine therapy	73 (77.6)	4 (57.1)	0.22
Anti-aromatase	41 (56.2)	4 (100)	0.08
Tamoxifen	32 (43.8)	0 (0)	

**Table 4:** Association between plaque development and cardiac structure exposure.

	All Patients		Left BC Patients	
Mean Doses, in Gy	Univariate Analysis OR at 1 Gy (95%CI)	*p*-Value	Univariate Analysis OR at 1 Gy (95%CI)	*p*-Value
**Any plaque development (N = 7)**				
Heart	1.28 (0.76–2.18)	0.35	1.07 (0.59–1.98)	0.81
LV	1.04 (0.85–1.28)	0.58	0.95 (0.74–1.22)	0.69
LAD	1.10 (0.99–1.21)	0.07	1.07 (0.97–1.20)	0.18
LM	**2.32 (1.08–4.97)**	**0.03**	**2.23 (0.99–5.05)**	**0.05**
CX	**2.27 (1.09–4.74)**	**0.03**	2.06 (0.93–4.53)	0.07
RCA	1.26 (0.36–4.40)	0.72	3.60 (0.66–19.67)	0.13
**Non-calcified plaque development (N = 4)**				
Heart	1.38 (0.69–2.77)	0.36	1.20 (0.55–2.63)	0.64
LV	1.06 (0.81–1.38)	0.66	0.98 (0.71–1.34)	0.89
LAD	1.11 (0.97–1.26)	0.12	1.09 (0.95–1.26)	0.22
LM	**2.77 (1.16–6.62)**	**0.02**	**2.72 (1.10–6.72)**	**0.03**
CX	**2.55 (1.03–6.29)**	**0.04**	2.37 (0.91–6.16)	0.07
RCA	1.26 (0.25–6.27)	0.78	3.26 (0.43–24.51)	0.25
**Calcified plaque development (N = 5)**				
Heart	1.23 (0.67–2.28)	0.51	1.03 (0.51–2.10)	0.93
LV	1.05 (0.83–1.33)	0.69	0.96 (0.72–1.28)	0.79
LAD	1.07 (0.97–1.20)	0.17	1.06 (0.94–1.20)	0.37
LM	**2.62 (1.15–5.97)**	**0.02**	**2.56 (1.07–6.10)**	**0.03**
CX	**3.03 (1.29–7.04)**	**0.01**	**2.84 (1.17–6.89)**	**0.02**
RCA	1.18 (0.26–5.40)	0.83	2.98 (0.45–20.04)	0.26

LM: Left main coronary artery; LAD: left anterior descending artery; LCX: left circumflex artery; RCA: right coronary artery.

## Data Availability

The datasets used and/or analyzed during the current study are available from the corresponding author on reasonable request.

## Appendix A

**Table A1:** Correlation matrix of mean doses to cardiac sub-structures.

	Heart	LV	LM	LAD	CX	RCA
**Heart**	1.00					
**LV**	**0.87**	1.00				
**LM**	**0.56**	**0.40**	1.00			
**LAD**	**0.81**	**0.77**	**0.43**	1.00		
**CX**	**0.55**	**0.52**	**0.73**	**0.44**	1.00	
**RCA**	0.05	−0.17	**0.30**	−0.15	−0.09	1.00

LV: Left Ventricle; LM: Left Main coronary artery; LAD: Left Anterior Descending artery; CX: Circumflex coronary artery; RCA: Right Coronary artery. Statistically significant correlation Pearson coefficients in bold. Blue cardiac structures are well correlated with each other (r^2^ > 0.70); Orange cardiac structures are well correlated with each other (r^2^ > 0.70).

**Table A2:** Description of localization of plaques and among the N = 7 patients with atherosclerotic plaque development.

Coronary Artery ^1^	LM	LAD						CX			RCA			
Patient\Segment	5	6	7	8	9	11	10	12	13	14	1	2	3	4
DJ070														
OE050														
MM003														
GM007														
TA048														
BA068														
MI110														

Emergence of new plaque.  Existent plaque. ^1^ LM: Left Main, LAD: Left anterior descending, CX: Circumflex, RCA: Right coronary artery. See Figure 1 for segment numbering.
